# Rare Oral Manifestations of Systemic Amyloidosis: A Case Report

**DOI:** 10.1155/crh/2622648

**Published:** 2025-10-06

**Authors:** Sizar Barazanji, Jair Gutierrez Herrera, Karin Garming-Legert

**Affiliations:** ^1^Karolinska Institute, Stockholm, Sweden; ^2^Karolinska University Laboratory, Stockholm, Sweden

## Abstract

This case illustrates the importance of medical and dental healthcare awareness of how amyloidosis may affect the oral mucosa. Careful, thorough examinations, and anamnesis in a stepwise manner, with communication between professions, are necessary to ensure an early diagnosis and avoid unnecessary suffering. In this case, the patient's medical history should have been taken into consideration when examining the oral cavity, as it might have been an indicator for the healthcare providers as to which tests were needed.

## 1. Introduction

Systemic immunoglobulin light chain (LC) amyloidosis is a nonproliferative protein misfolding disease where fragments of immunoglobulin LCs' aggregate in tissues that can lead to organ dysfunction [1, 2]. The protein deposits mechanically disrupt several organ tissues, producing a cytotoxic, proapoptotic effect [3].

Amyloidosis is considered a rare disease with an annual incidence of approximately 12 per million in the United States and European Union [2, 4]. Previous studies conducted in Sweden have reported an incidence of 8.29 per million persons per year, with incidence slightly higher in men than that in women [5]. Amyloidosis varies widely depending on its subtype and has a low specificity. Amyloid light-chain amyloidosis (AL amyloidosis) is considered the most frequently occurring form [3]. In AL amyloidosis, the LC proteins, which normally conform to an α-helical structure, instead misfold into β-pleated sheets [2]. These misfolded proteins become visible after positive staining with Congo red when viewed under polarized light in a histopathological exam. A tissue biopsy is compulsory to demonstrate the presence of amyloid proteins [2, 4, 6, 7].

AL amyloidosis progresses rapidly, and the 4-year survival rate is estimated to be between 40% and 60% [3]. The survival rate is usually dependent on the extent of the interference with any critical organ function [5, 7]. In particular, the extent of cardiac involvement is a major determinant of the outcome of amyloidosis [2]. Amyloid protein can deposit in various locations, and it is usually the mechanical effect of the deposits that causes the organs to dysfunction, for example, a stiffening of the cardiac muscle, which can occur in advanced cardiac amyloidosis [7, 8].

Multiple myeloma (MM) is also a clonal plasma cell disorder, and AL amyloidosis can commonly be found MM while the opposite is less frequent. Around 10%–15% of patients with MM have AL amyloidosis. Nevertheless, data on both incidence and survival rate of these patients are limited. Slightly more males than females with MM are diagnosed with AL amyloidosis, except for secondary systemic amyloidosis where incidence is higher in females. AL amyloidosis, in both male and female patients, is rarely diagnosed below age 65 years [5, 7, 9].

Intraorally, the manifestations of amyloid protein deposits commonly affect the tongue. This appears clinically as macroglossia with pronounced dental impressions of the dental arch on the tongue. In some cases, macroglossia might even be severe enough to cause difficulties in speaking and chewing [4, 7, 10, 11]. However, macroglossia occurs in 15% of AL amyloidosis cases [7].

## 2. Case Presentation

**Figure 1:**
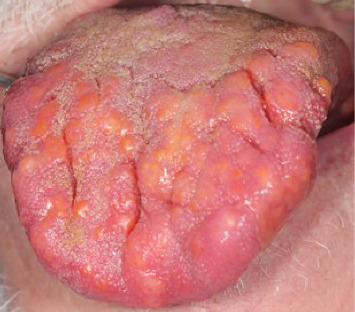
Oral clinical findings upon visit at the dental clinic. Yellow and orange nodular lesions in varying size and shape found on the dorsal surface of the tongue. Overall macroglossia of the entire tongue with fissures more prominently on the dorsal surface of the tongue as well as irregular lateral borders due to the enlargement of some of the nodules.

**Figure 2:**
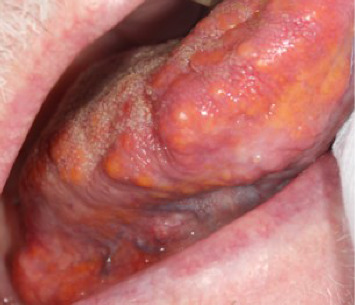
Oral clinical findings upon visit at the dental clinic. Yellow and orange nodular lesions in varying size and shape found on the right surface of the tongue. Overall macroglossia of the entire tongue with fissures more prominently on the dorsal surface of the tongue as well as irregular lateral borders due to the enlargement of some of the nodules.

**Figure 3:**
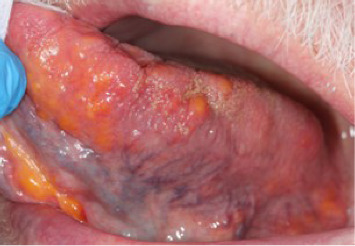
Oral clinical findings upon visit at the dental clinic. Yellow and orange nodular lesions in varying size and shape found on the left surface of the tongue. Overall macroglossia of the entire tongue with fissures more prominently on the dorsal surface of the tongue as well as irregular lateral borders due to the enlargement of some of the nodules.

**Figure 4:**
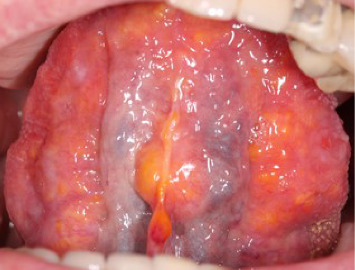
Oral clinical findings upon visit at the dental clinic. Yellow and orange nodular lesions in varying size and shape found on the ventral surface of the tongue. Overall macroglossia of the entire tongue with fissures more prominently on the dorsal surface of the tongue as well as irregular lateral borders due to the enlargement of some of the nodules.

A 76-year-old male sought medical health care for perceived painful ulcerations and blisters on the tongue and palate. The patient had a medical history of high blood pressure, third stage chronic kidney disease, gonarthrosis, and malignant tumor in the prostate. The patient reported an involuntary weight loss of 2 kg and fatigue 2 weeks prior to the visit, which he believed to be a cause of the painful condition he was experiencing in the oral cavity. The first clinical examination by a general practitioner observed different sized and colored coatings, and nodular, bulbous, and hemorrhagic lesions on the dorsal and ventral surfaces of the tongue (see Figures 1, 2, 3, and 4). No primary diagnosis was possible, and the patient was referred to an ear, nose, and throat (ENT) specialist.

A punch biopsy of the tongue taken during the clinical examination at the ENT clinic revealed what was suspected to be a hemangioma and oral candidiasis. The patient was prescribed local treatment for oral candidiasis due to the symptoms he was experiencing; however, no improvement had occurred at the 4-week control. During this time, the patient also sought help for weakness in the hands and pain in the shoulders and arms. The clinic then referred the patient to another ENT specialist at a larger hospital. Their oral examination recorded the same clinical findings on all surfaces of the tongue, the floor of the mouth, and the frenulum of the tongue.

A rheumatologist was consulted to see if the patient history of kidney failure and joint pains had any connection with the oral findings. Testing of antinuclear antibodies and antineutrophil cytoplasmic antibodies was done to exclude systemic lupus erythematosus and scleroderma; however, the results were negative.

**Figure 5:**
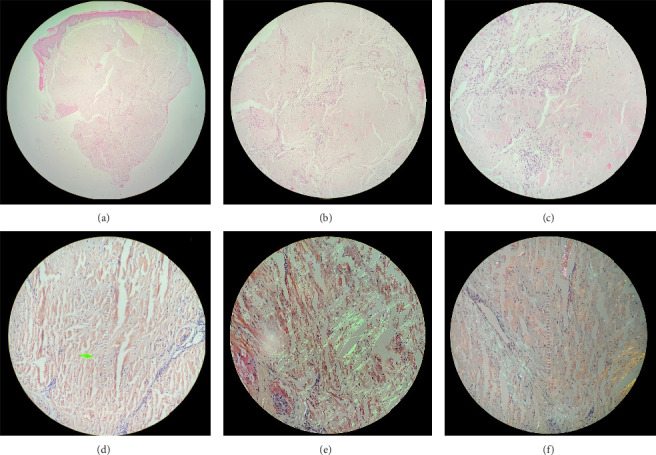
(a) Tongue biopsy with hematoxiline & eosine stain at 4x magnification. (b) Same image at 10x magnification; eosinophilic bundles are now visible in the middle of fibrous stroma and some vessels. (c) Higher magnification (20x) showing inflammatory cells around the amorphous and compact bundles. (d) Same area as in (c), but stained with congo red, at 20x magnification without polarization (see tip of the green arrow). (e) Prevalence of amyloidosis is observed in the stroma and around some of the vessels with typical green-apple staining after polarization at 40x magnification. (f) This is also evident around the vessels at 40x magnification.

The first biopsy of the tongue was re-evaluated with a suspicion of sarcoidosis or malignancy, but the second evaluation had no signs of granuloma and no markers to confirm the suspicion of sarcoidosis, and the results were inconclusive due to lack of sample material. With all other preliminary diagnoses excluded, a new biopsy of the tongue of the patient was taken with the suspicion of amyloidosis due to the patient's other health problems being taken into consideration. The tissue sample was stained with Congo red and viewed under a polarized light microscope; the apple-green birefringence that was visible confirmed a preliminary diagnosis of amyloidosis (see Figure 5). Further evaluation revealed abundant AL amyloid lambda, confirming the AL amyloidosis subtype. Congo-red staining of a bone-marrow biopsy confirmed the diagnosis. To determine whether the amyloidosis was systemic or localized, an abdominal fat biopsy was taken. The presence of an abundance of amyloid-protein aggregates confirmed the systemic nature of the amyloidosis.

AL amyloidosis produces free monoclonal LCs in the urine, called Bence Jones proteins of the κ or λ isotype, such as MM. The difference, however, is that while κ LCs are more prevalent in MM, the ratio of κ-to-λ LCs in AL amyloidosis is 1:3 [12]. A MM diagnosis, however, requires the presence of one or more myeloma defining events (MDE), such as renal insufficiency, increased marrow clonal plasma cells, increased serum free LCs, anemia, bone lesions, and hypercalcemia [13]. The patient had a bone marrow biopsy taken due to continued skeletal pains, 3 months after the amyloidosis diagnosis was confirmed. Previous serum testing had found no M-component but free λ LCs. The histopathological results of the bone marrow, however, showed amyloidosis deposits after Congo-staining, and an increase in clonal plasma cells (50%). The conclusive diagnosis was then confirmed to be systemic amyloidosis as well as MM; this is due to the presence of several MDE features that the patient had, such as renal insufficiency, anemia, and increased clonal plasma cells in bone marrow.

### 2.1. Differential Diagnosis

The oral manifestations of amyloidosis as previously discussed may present themselves with nonspecific symptoms such as macroglossia. However, given the clinical presentation of this case, the differential diagnoses that were discussed were hemangioma, sarcoidosis, tumors of the oral cavity, or lymphoid hyperplasia. These were however all ruled out due to the last tissue biopsy of the tongue.

### 2.2. Clinical and Histopathological Findings

Oral clinical findings upon visit at the dental clinic. Yellow and orange nodular lesions in varying size and shape found on the dorsal (1), right (2) and left (3) lateral borders, and ventral (4) surface of the tongue. Overall macroglossia of the entire tongue with fissures more prominently on the dorsal surface of the tongue as well as irregular lateral borders due to the enlargement of some of the nodules.

In Figure 5(a), tongue biopsy with Hematoxylin & Eosine stain at 4x magnification.  5(b), same image at 10x magnification; eosinophilic bundles are now visible in the middle of fibrous stroma and some vessels. In Figure 5(c), higher magnification (20x) show inflammatory cells around the amorphous and compact bundles. In Figure 5(d), same area as in 5(c), but stained with Congo red, at 20x magnification without polarization (see tip of the green arrow). In Figure 5(e), prevalence of amyloidosis is observed in the stroma and around some of the vessels with typical green-apple staining after polarization at 40x magnification. In Figure 5(f), this is also evident around the vessels at 40x magnification.

## 3. Discussion

In this case, we cannot conclude whether the initial oral lesions examined were the same lesions which raised the suspicion of amyloidosis at our clinic since the primary physician's office archived no clinical images from the first examination. However, what this report wishes is to heighten awareness, on the part of the dental caregiver, of how important it is to do a thorough clinical examination: Many systemic diseases present with oral manifestations, and in some of these cases, the earlier the diagnosis, the better the prognosis [14].

As mentioned, a histopathological examination is essential to diagnose AL amyloidosis, and for this, a tissue biopsy is required. The oral cavity is an area that is easily accessible for both examination and tissue biopsy. Thus, the oral mucosa can be a viable substitute for kidney tissue or abdominal fat, biopsies of which are more invasive and can cause the patient more discomfort [12]. The diagnostic delay due to the unclear nature of the disease, however, is one of the biggest obstacles in the patient care of amyloidosis. An awareness of amyloidosis and the means for early diagnosis are thus of utmost importance for a better prognosis [8]. The initial biopsy taken by the first ENT specialist had no tentative diagnosis and showed “squamous epithelium with reactive changes in the form of spongiosis and exocytosis, with a slight fibrotic stroma in the subepithelial region with an increased number of slightly slit-shaped vascular areas.” The suspected first pathological finding was a hemangioma, but the second evaluation could not confirm the initial one, and it was therefore ruled out.

One of the most common oral manifestations of AL amyloidosis is macroglossia, which can be debilitating for the patient. In this case, the patient was experiencing difficulties in normal oral functions such as speaking, chewing, swallowing, and maintaining oral hygiene due to discomfort caused by the tongue enlargement as well as a sense of self-consciousness about the appearance of their tongue. The enlarged tongue can also develop deep dental impressions, which can be further sources of irritation [15]. Dental health practitioners can support the patient through treatments that improve oral hygiene, by manufacturing a soft bite rail to reduce friction, and in some cases, prescribing an analgesic mouth rinse or gel.

In recent decades, treatment of AL amyloidosis has advanced, and chemotherapy is now the main treatment. Developments in chemotherapy now target the suppression of the plasma cell clones that secrete Ig LCs, which form amyloid. When a treatment causes the amyloidogenic proteins to decrease, a catabolic reaction to the deposits of amyloid in the tissues occurs. As a consequence, the release of free LCs with subsequent amyloid deposits in organ tissue slows down, or stops altogether [8]. Similar treatments have shown to be effective against lymphoproliferative diseases and MM. The overall survival rate of AL amyloidosis was considered poor in the 90s, with an average of once only 18 months. The prognosis of the disease in the 21st century, however, has improved with treatments that successfully suppress LC formation [7].

Daratumumab, a monoclonal antibody that targets antigens such as IgG, is a generally well-approved subcutaneous infusion which has demonstrated a deep hematological response, sometimes after the first infusion. It is now generally considered the treatment of choice for patients who are 65 years or older, who have decreased kidney function (eGFR ≤ 50 mL/min), when more than two organs are involved, or any combination of these factors. This is true for patients with MM, AL amyloidosis, or both [3, 8, 16].

### 3.1. Outcome and Follow-Up

Treatment with Daratumumab, a monoclonal anti-CD38 antibody that targets antigens expressed on myeloma cells, was begun. A few months after starting this treatment, the patient experienced some improvement in fatigue and in general health. No long-term follow-up has been possible on this patient, so the effect of the treatment on the oral manifestations is unknown.

## Data Availability

All data used for this case report are contained within the article and its supporting files. These data are freely available for use without restriction.
